# A Micro-Test Structure for the Thermal Expansion Coefficient of Metal Materials

**DOI:** 10.3390/mi8030070

**Published:** 2017-02-28

**Authors:** Qingying Ren, Lifeng Wang, Qingan Huang

**Affiliations:** Key Laboratory of MEMS of the Ministry of Education, Southeast University, Nanjing 210096, China; seurqy@163.com (Q.R.); wanglyfeng@hotmail.com (L.W.)

**Keywords:** cascaded chevrons, thermal expansion coefficient, thermal drive method, extraction method, metal materials

## Abstract

An innovative micro-test structure for detecting the thermal expansion coefficient (TEC) of metal materials is presented in this work. Throughout this method, a whole temperature sensing moveable structures are supported by four groups of cascaded chevrons beams and packed together. Thermal expansion of the metal material causes the deflection of the cascaded chevrons, which leads to the capacitance variation. By detecting the capacitance value at different temperatures, the TEC value of the metal materials can be calculated. A finite element model has been established to verify the relationship between the TEC of the material and the displacement of the structure on horizontal and vertical directions, thus a function of temperature for different values of TEC can be deduced. In order to verify the analytical model, a suspended-capacitive micro-test structure has been fabricated by MetalMUMPs process and tested in a climate chamber. Test results show that in the temperature range from 30 °C to 80 °C, the TEC of the test material is 13.4 × 10^−6^ °C^−1^ with a maximum relative error of 0.8% compared with the given curve of relationship between displacement and temperature.

## 1. Introduction

Successful design of a microelectromechanical systems (MEMS) device should take into consideration electrical engineering, mechanical engineering, material processing and microfabrication. In the mechanical design stages, the thermal expansion coefficient (TEC) of the metal structure is one of the most critical properties to be considered [1], which will be directly linked to the flexibility of the support beams and the dynamic characteristics, and further affect the performance of the MEMS devices. For example, most of the temperature-driven MEMS devices are based on thermal expansion effect [2,3], and mismatch of the TECs between bimetallic cantilevers may result in low reliability of micro devices [4,5]. In addition, the TEC of metal structure also gives useful information about materials’ electronic and magnetic properties [6], their specific heat, as well as thermodynamic phase transitions [7]. The TEC of a certain material may vary with the MEMS fabrication process under different manufacturing conditions [8]. Therefore, it is essential to confirm the TEC of metal materials to improve or predict the performance of MEMS devices, and it can also provide direct quality-control information for the fabrication process line [9]. 

There are a lot of available techniques can be utilized to measure the TEC of metal materials, e.g., current-driven vernier microgauge methods [10,11], optical images and diffraction patterns methods [12,13], as well as *x*-ray diffraction methods [14], etc. Compared with those techniques, capacitance methods are preferable since their higher sensitivity, simpler operation and more flexible readout process [15,16,17]. In this work, a micro-test suspended-capacitive metal structure has been proposed to determine the TEC in the temperature range from 30 °C to 80 °C. The novel micro-test structure model has been described in details and analyzed theoretically, while a finite element software is then utilized to verify the relationship between the TEC of the material and displacements of the structure in horizontal and vertical directions. A suspended-capacitive structure fabricated by MetalMUMPs process has been measured in a climate chamber, with a TEC value of (13.4 ± 0.1) × 10^−6^ °C^−1^.

## 2. Test Structure and Analytical Model

**Figure 1:**
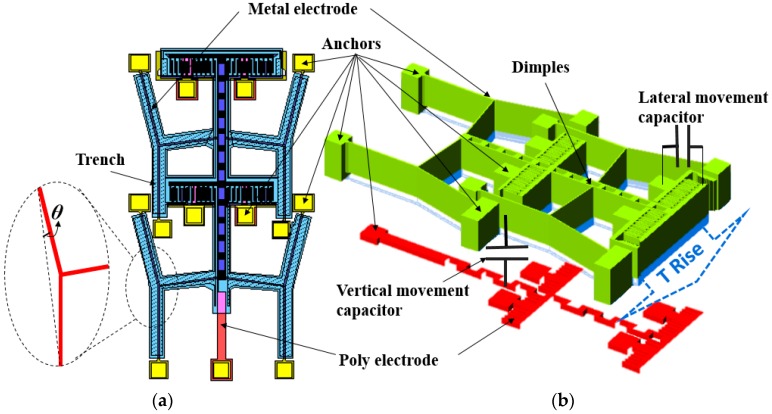
The novel thermal expansion coefficient (TEC) micro-test structure. (**a**) The main top-view layouts of the suspended capacitor structure, four groups of cascaded V-shaped beam deformation will cause displacements of the moveable electrodes in horizontal and vertical directions, respectively; (**b**) 3D configuration of suspended capacitor, which consists of lateral and vertical temperature movement sensitive part.

This capacitive TEC measurement structure is designed based on a micro-thermal actuator configuration, as shown in Figure 1. There is a temperature sensing capacitive structure which is fabricated by the MetalMUMPs process. In Figure 1a, four groups of cascaded chevrons are packed close together and linked with the temperature sensing moveable capacitors. Thermal expansion of the metal material causes the deflection of the cascaded chevrons, which leads to the capacitance variation. Figure 1b describes the detail information about the suspended-capacitive structure that consists of two capacitors. The lateral movement capacitor consists four pairs of interdigital capacitors, which are constructed by the metal layer, and the vertical movement capacitor is fabricated by the metal and poly layers. Thermal expansion of the chevron beams induces the capacitance variations on vertical and horizontal directions, which can be recognized as the displacements of the moveable electrodes on both directions. Thus, by detecting the capacitance value of temperature sensing capacitors at different temperatures, the TEC values of the metal materials can be deduced and extracted correspondently. 

### 2.1. Theory Model of Vertical Sensing Capacitor

The vertical sensing capacitor changing with temperature is mainly caused by the thermal expansion of Si substrate, which will introduce thermal expansion mismatch in metal Ni layers, and further lead to the thermal deflection of the metal anchor. As the metal anchor film is much thinner than the Si substrate, a good approximation to assume that the thin film anchor contracts according to the substrate’s TEC [18]. The only way the film can achieve the state is to develop an in-plane biaxial inner strain, which is the thermal mismatch strain in metal Ni, and can be given by

(1) $$\sigma_{\mathrm{Ni},\mathrm{mismatch}}=\left(\frac{E}{1-\mu }\right)\left(\alpha_{\mathrm{Ni}}-\alpha_{\mathrm{Si}}\right)\left(T-T_{0}\right),$$

where *E* and μ are the Young’s modulus and Poisson ratio of the metal Ni, respectively. *T*_0_ represents the initial temperature. Because of the Poisson effect, the vertical strain has an additional out-of-plane strain set up by the in-plane thermal-mismatch strain. Hence, the total out-of-plane strain in the anchor is

(2) $$\sigma_{\mathrm{Ni},z}=\left(\frac{E}{1-\mu }\right)\left[\alpha_{\mathrm{Ni}}+2\mu \left(\alpha_{\mathrm{Ni}}-\alpha_{\mathrm{Si}}\right)\right]\left(T-T_{0}\right)$$

**Figure 2:**
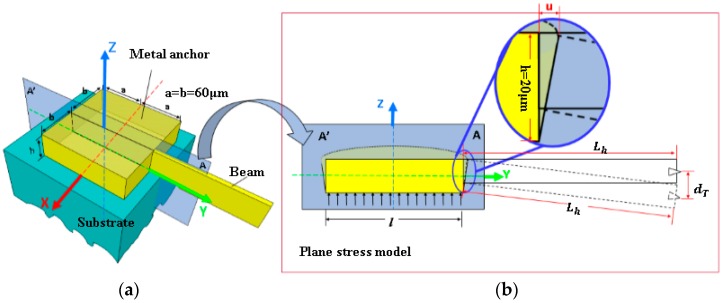
Structure of the metal anchor on the silicon substrate. (**a**) Beam end posted anchor model with the coordinate axes; (**b**) beam deflection caused by the anchor thermal deformation in the plane stress model.

A CAD model for a typical anchor is shown in Figure 2 with a buckle-beam cross-section shown in Figure 2b. As the metal film is thin in the *z* direction, then the plate with the *yz*-plane as its middle plane. In this way, we can simplify problems of thermal stress in the anchor into problems of plane stress. Taking into account the principle of stress-strain relations and Hooke’s law [19], the displacement of each point in *y* directions can be given by

(3) $$u_{\mathrm{total}}=\int \text{​}\left(\alpha_{\mathrm{Ni}}-\alpha_{\mathrm{Si}}\right)\left(T-T_{0}\right)dy-\frac{1}{h}\frac{\mu yz\left(\alpha_{\mathrm{Ni}}-\alpha_{\mathrm{Si}}\right)\left(T-T_{0}\right)}{1-\mu }$$

where the thickness *h* of metal Ni is 20 μm, the length of the half anchor is 60 μm. Considering the geometric relation as shown in Figure 2, *L_h_* is the half length of the effective length between two anchors. Thermal deflection of the metal Ni anchor will introduce a vertical displacement of the beams tip as $d_{T}$, which can be given by

(4) $$\begin{array}{ll} d_{T} & =\frac{u_{\mathrm{total}}}{h}L_{h}{|}_{y=60\mu m，z=h=20\mu m}\\ & =\frac{h-\left(h+z\right)\mu L_{h}}{h^{2}\left(1-\mu \right)}\left(\alpha_{\mathrm{Ni}}-\alpha_{\mathrm{Si}}\right)\left(T-T_{0}\right)y{|}_{y=60\mu m，z=h=20\mu m}\\ & =\frac{3\left(1-2\mu \right)L_{h}}{1-\mu }\left(\alpha_{\mathrm{Ni}}-\alpha_{\mathrm{Si}}\right)\left(T-T_{0}\right) \end{array}$$

**Figure 3:**
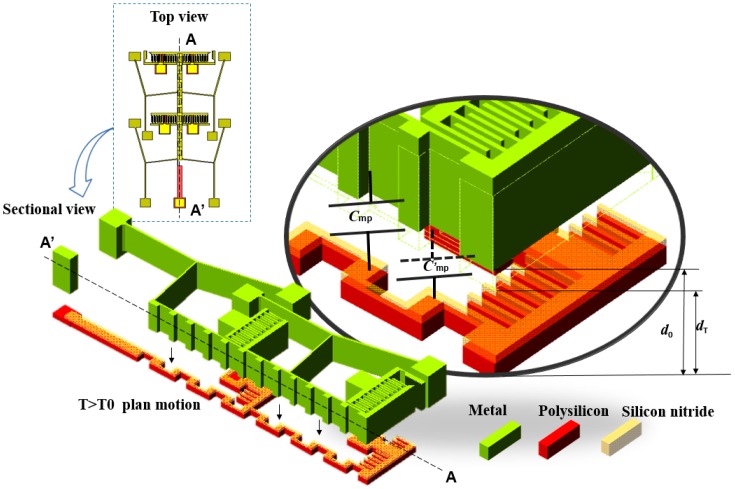
Sectional view of the vertical movement capacitor, displacement in the vertical direction will vary with temperature. The metal nickel layer is designed as the top electrode of the capacitor, and it is a movable center shuttle supported by four groups of cascaded V-shaped beams anchored at their ends. Poly layer is designed as the bottom electrode of the vertical movement capacitors, which has a silicon nitride isolation layer and an air gap over it.

The equation indicates that the vertical displacement of the tip linked layer linearly dependence on rising temperature. For the vertical capacitor, as indicated in Figure 3, $d_{0}$ is the initial distance between the two electrodes layer. When the temperature changes, the top layer will move and induce a variation in the sensing capacitor, and the vertical capacitance changes with temperature varies can be expressed as

(5) $$C_{\mathrm{mp}}\left(T\right)=\frac{\varepsilon S}{d_{0}-|d_{T}|}$$

where *S* is the area of overlap of the electrode layer and poly electrode layer. From Equations (4) and (5), it shows that the TEC of the metal material can be given by

(6) $$\alpha_{\mathrm{Ni},\mathrm{mp}}=\frac{1-\mu }{3\left(T-T_{0}\right)\left(1-2\mu \right)L_{h}}\left[d_{0}-\frac{\varepsilon S}{C_{\mathrm{mp}}\left(T\right)}\right]+\alpha_{\mathrm{si}}$$

### 2.2. Theory Model of Lateral Sensing Capacitor

**Figure 4:**
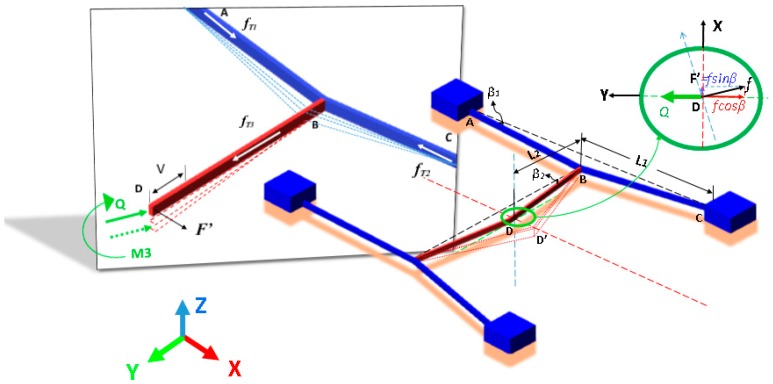
Half span of the cascaded V-shaped beam structure: geometry and loads.

In order to get a satisfied horizontal displacement, two groups of cascaded V-shaped beam structure are designed. Figure 4 shows the half span of the cascaded V-shaped test structure, axial force *f_T_* = *f_T_* = *f_T_*_1_ = *f_T_*_1_ = α_Ni_*AE*(*T* − *T*_0_) and the angle β = β_1_ of beam have been indicated in figure, *A* and *L* are the cross-sectional area and length of the beam, respectively. According to geometric symmetry, force, and moment equilibrium conditions, the reaction forces acting at the anchor of the beam can be expressed as

(7) $$F^{\prime }=f\sin\beta =\left(f_{T1}{\sin\beta }_{1}+f_{T2}{\sin\beta }_{1}+f_{T3}\right)\sin\beta =f_{T}\left(2\sin\beta +1\right)\sin\beta$$

The beam bending moment is

(8) $$M=F^{\prime }\cos\beta v=f_{T}\left(2\sin\beta +1\right)\sin\beta \cos\beta v$$

(9) $$\frac{\partial M}{\partial F^{\prime }}=\cos\beta v$$

According to Castigliano’s second theorem [20], the displacement along the horizontal direction can be obtained as

(10) $$\begin{array}{cc} \delta_{x} & =\frac{\partial U}{\partial F^{\prime }}=\frac{1}{EI}\int_{0}^{L}M\frac{\partial M}{\partial F^{\prime }}dv=\frac{1}{EI}\int_{0}^{L}f_{T}\left(2\sin\beta +1\right){\sin\beta \cos}^{2}\beta v^{2}dv\\ & =\frac{\alpha_{\mathrm{Ni}}AE\left(T-T_{0}\right)}{3EI}\left(2\sin\beta +1\right){\sin\beta \cos}^{2}\beta \end{array}$$

where moment of inertia is *I* = *I_xx_* + *I_yy_* with

$$I_{xx}=\int_{-L/2}^{+L/2}\frac{A}{L}u^{2}\sin^{2}\beta du=\frac{AL^{3}}{12}\sin^{2}\beta ,$$

$$I_{yy}=\int_{0}^{L}A\xi^{2}d\xi =\frac{AL^{3}}{3}\cos^{2}\beta .$$

**Figure 5:**
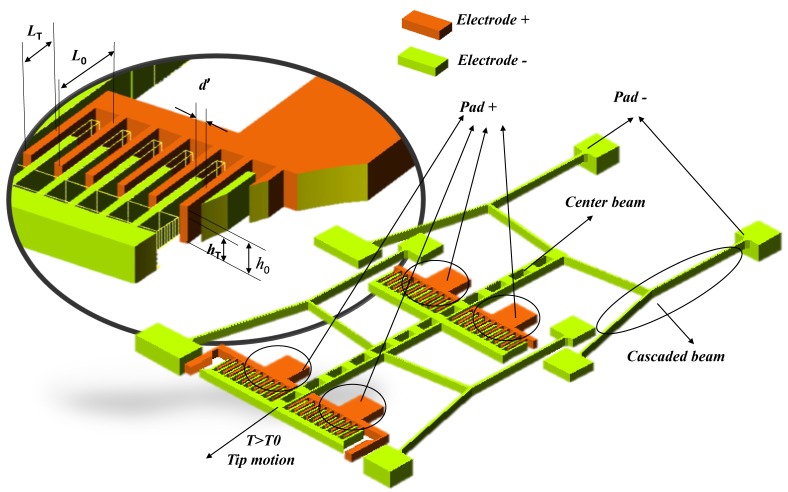
Configuration of the lateral movement capacitor, it consists of four groups of interdigital electrodes. Four groups of cascaded V-shaped beam deformation with vary temperature will cause displacements of the moveable electrodes in the horizontal and vertical directions.

As shown in Figure 5, the lateral movement capacitor consists of four groups of interdigital electrodes, h and $L_{0}$ are the height and length of overlap in initial temperature $T_{0}$, *d*’ is the distance between the electrodes. $L_{T}=L_{0}$ − $\delta_{x}$ is the length of overlap in temperature T. Lateral capacitance changes with temperature varies can be expressed as

(11) $$C_{mm}\left(T\right)=\varepsilon \frac{h_{T}L_{T}}{d^{\prime }}\;=\varepsilon \frac{h\left(L_{0}-\delta_{x}\right)}{d^{\prime }}$$

From Equations (10) and (11), TEC of the metal material with temperature can be solved by

(12) $$\alpha_{\mathrm{Ni},mm}=\frac{3EI\left(\varepsilon hL_{0}-C_{mm}\left(T\right)d^{\prime }\right)}{\varepsilon hAE\left(T-T_{0}\right)\left(2\sin\beta +1\right){\sin\beta \cos}^{2}\beta }$$

## 3. Finite Element Simulations

**Figure 6:**
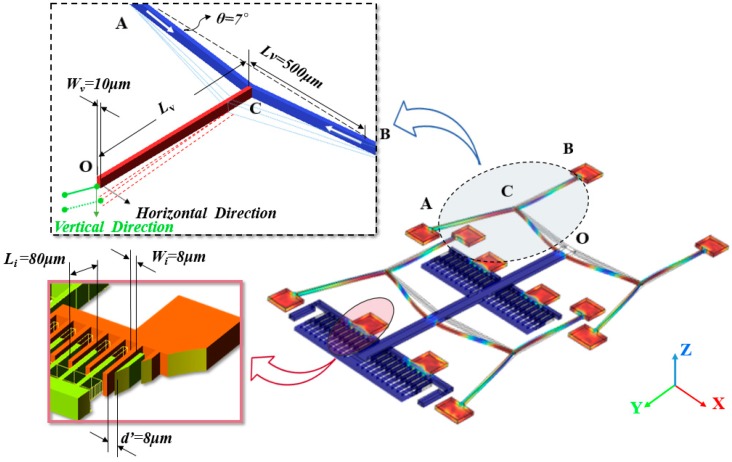
Detail metal layer information about the test structure parameters for the finite element simulations. The polysilicon layer has the same structure of the center beam and four groups of interdigital electrodes. Stress distribution has been plotted in color temperature.

In this section, a finite element model has been established to verify the relationship between the thermal expansion of the material and displacements of the structure in the horizontal and vertical direction. The parameters for the simulation are listed in Table 1, and the test structure parameters for the finite element simulations are shown in Figure 6. As discussed in section one, the thin metal film anchor contracts according to the substrate’s thermal expansion coefficient, and there is an additional thermal mismatch strain in metal anchor. In the simulate model, the thermal expansion coefficient of anchors is set as TEC of Si, and the inner strain is set as Equation (2) with fixed boundary. During the finite element simulation process, initial temperature of the test structure is assumed at room temperature 20 °C, environment temperature has been set from 30 °C to 80 °C with a constant step of 10 °C. The capacitance of test structure changes with temperature in vertical direction and horizontal directions, which agrees well with the prediction of the analytical model.

**Figure 7:**
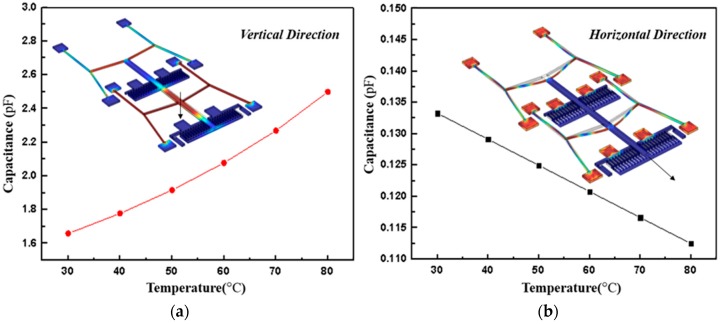
The finite element simulation results. (**a**) The variation of the vertical direction sensing capacitance versus temperature; (**b**) the sensing capacitance varies with temperature in the horizontal direction.

**Figure 8:**
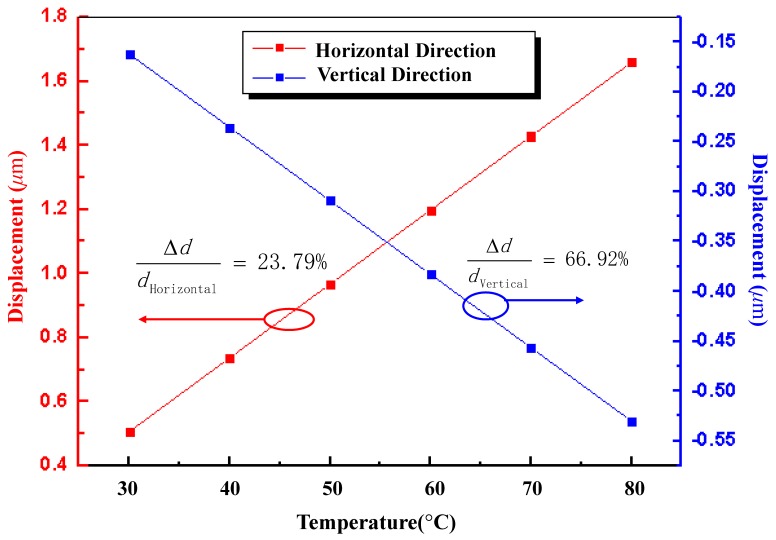
Comparison of the displacement variation with temperature in the horizontal and vertical directions. Both directions demonstrate high-linearity output results. Changing rate of displacements with temperature in vertical direction is about three times higher than that in the horizontal direction.

Figure 7 provides the simulation results of the changing capacitance as a function of temperature. In the whole test temperature range, the capacitance modification is ~0.84 pF with the initial value is 1.66 pF, and the relative change capacitance of horizontal capacitor is 0.02 pF with the initial value is about 0.13 pF. It can be concluded that, for given geometry parameters, the test structure in the vertical direction has higher sensitivity than that in the horizontal direction. However, the changes of capacitance with temperature have better linearity in the horizontal direction. To better show the relationship between test structure and TEC of the material, displacements changing with temperature in the horizontal and vertical directions are also investigated as demonstrated in Figure 8. Changing rate of displacements with temperature in the vertical direction is about 66.92%, which is about three times higher than that in the horizontal direction, and both directions demonstrate high-linearity output results.

## 4. Fabrication and Experiment

### 4.1. Fabrication

**Figure 9:**
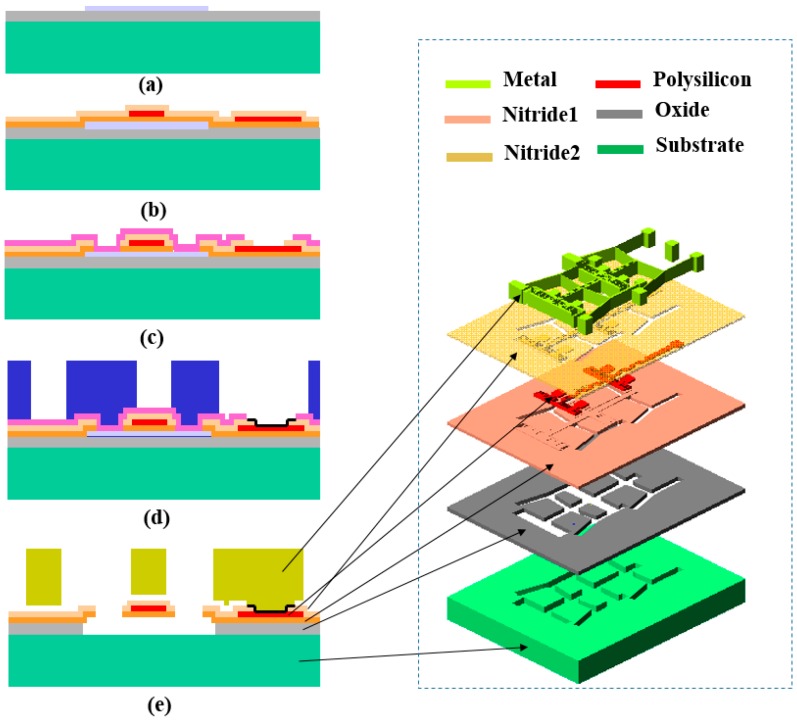
Fabrication process of the micro-test capacitive structure. Standard processing service was provided by MetalMUMPs (MEMSCAP Co., Durham, NC, USA). The briefing fabrication steps are listed on the left-hand part from cross-sectional view, whilst the detached vertical layer are plotted on the right side, separately.

A micro-test structure for detecting the TEC of metal materials is presented to verify the analytical model. The test structure is fabricated using MetalMUMPs process. MetalMUMPs is a standard electroplated nickel micromachining process, which is suitable for fabrication of MEMS metal devices, as shown on Figure 9. Firstly, two oxide layers are patterned and served as sacrificial layers (Figure 9a). Then, silicon nitride layer (nitride 1) is deposited, immediately following by a deposition process of polysilicon layer. After the polysilicon is lithographically patterned, a second silicon nitride layer (nitride 2) will be deposited (Figure 9b). The combined nitride layers (nitride 1 and nitride 2) provide a protective encapsulation for the 0.7 µm thickness doped polysilicon that is used as the electrode pads of the vertical capacitor and electrical routing connection of the test structure. In the following step, reactive ion etching (RIE) is performed to pattern nitride layers and another sacrificial layer will be deposited (Figure 9c). Afterwards, anchor metal and plating base layer are patterned on the surface layer, while a thick layer of photoresist is patterned and deposited to form the stencil of the electroplated metal layer (Figure 9d). Finally, a metal nickel layer with 20 µm thickness is electroplated to nominal thickness, and meanwhile, served as the top electrode material and electrical interconnect layer. After removing the photoresist stencil and plating base, the sacrificial layers will be released to form the air gap between polysilicon layer and nickel layer. A trench in the substrate is created after the isolation oxide releasing process. The trench can improve the test structure performance by providing additional thermal and electrical isolation (Figure 9e).

**Figure 10:**
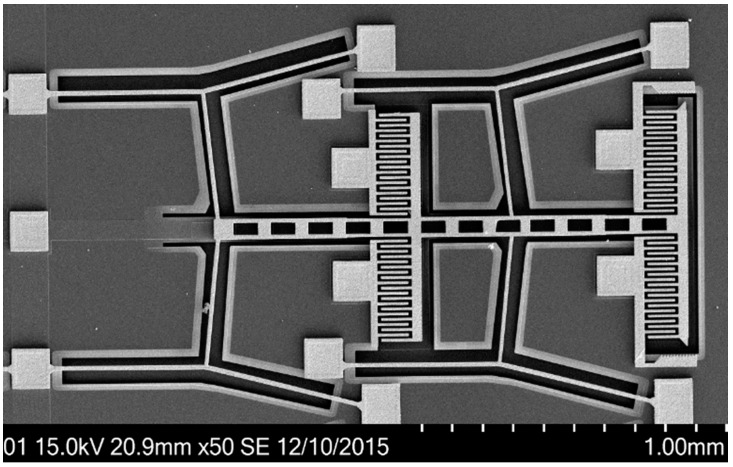
Scanning electron microscope (SEM) photographs of the suspended capacitor.

Figure 10 is the scanning electron microscope (SEM) image of the suspended temperature sensing capacitor showing some detailed microstructures. Four groups of chevron beams are packed close together and linked with the center moveable electrodes, the other electrode polysilicon layer of the vertical capacitor is located underneath. Four pairs of interdigital beams formed the lateral movement capacitors. Trenches in the substrate have been well released and the suspended capacitive structure remains consistently as initial designed.

### 4.2. Experiment and Discussion

**Figure 11:**
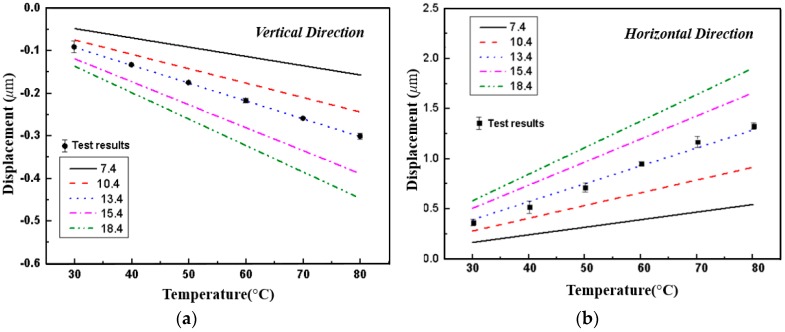
Theoretical and measured relationship between displacements versus temperature. (**a**) The variation of the vertical displacements versus temperature; (**b**) variation of the horizontal displacements versus temperature.

The test structure is measured inside a climate chamber. The temperature of the chamber is controlled at six temperature stages within a step of 10 °C ranging from 30 °C to 80 °C. To illustrate the measured results, Figure 9 gives the simulation results of displacement versus temperature with TEC values of 7.4 × 10^−6^ °C^−1^, 10.4 × 10^−6^ °C^−1^, 13.4 × 10^−6^ °C^−1^, 15.4 × 10^−6^ °C^−1^ and 18.4 × 10^6^ °C^−1^ in the vertical direction and horizontal direction, respectively. It is obvious that a greater TEC value indicates a higher changing rate correspondently, as illustrated in Figure 11. It can be concluded that the test result curves fit well with the curve while the TEC is 13.4 × 10^−6^ °C^−1^, and the maximum relative errors are 0.3% and 0.8% in the vertical and horizontal directions. Thus, from the theoretical/measurement contrast information, it is reasonable to conclude that test TEC of the metal materials is linear and creditable in the whole temperature range.

The source of error mainly comes from the approximations and assumptions in theoretical model and the measurement process. In theoretical model, the anchor thermal deformation analysis is simplified to the plane thermal stress model. In addition, the material parameters are assumed to be some constant over the test temperature range, which may not be true in real condition. On the other hand, there may have some fabrication geometrical deviation of the test structure itself, and the deviation of the measurement equipment can also bring the error in the final results. However, in further research, complementary test structures for other material parameters, such as residual stress and Young’s modulus, could be designed to meet the higher accuracy requirements. In addition, replicated basic test structure cells should be designed and connected in parallel to amplify the outputs signal and reduce the random error.

## 5. Conclusions

This paper presents a novel suspended-test structure for detecting the TEC of metal material. Theory model is established and analysis. Finite element model is used to verify the relationship between the TEC of the material and displacements of the structure in the horizontal and vertical directions. A suspended-capacitor structure fabricated by the MetalMUMPs process is tested in a climate chamber. Experimental results show that the new type of capacitive TEC micro-test structure can provide a stabilized measurement of TEC with the value of (13.4 ± 0.1) × 10^−6^ °C^−1^.

## Figures and Tables

**Table 1:** Parameters for the finite element simulations.

Parameter	Value
Young’s modulus E	2.19 × 10^11^ (Pa)
Poisson’s ratio μ	0.31
Thermal conductivity	90.7 (W/(m·K))
TEC of Si	2.33 × 10^−6^ °C^−1^
TEC of Ni	13 × 10^−6^ °C^−1^
Vacuum permittivity	8.85 × 10^−^^12^
Relative dielectric constant of free space ε	1
